# Comprehensive review of implications of COVID‐19 on clinical outcomes of cancer patients and management of solid tumors during the pandemic

**DOI:** 10.1002/cam4.3534

**Published:** 2020-10-20

**Authors:** Ankit Madan, Joshua Siglin, Aleem Khan

**Affiliations:** ^1^ Department of Internal Medicine SOVAH Cancer Center Danville VA USA; ^2^ Department of Radiation oncology Duke University Raleigh NC USA; ^3^ Department of Psychiatry Salem VA medical Center University of Virginia School of Medicine Salem VA USA

**Keywords:** cancer, COVID‐19, oncology, outcomes, pandemic, recommendations, review article

## Abstract

Coronavirus disease‐2019 (COVID‐19) has emerged as a novel infection which has spread rapidly across the globe and currently presents a grave threat to the health of vulnerable patient populations like those with malignancy, elderly, and immunocompromised. Healthcare systems across the world are grappling with the detrimental impact of this pandemic while learning about this novel disease and concurrently developing vaccines, strategies to mitigate its spread, and treat those infected. Cancer patients today face with a unique situation. They are susceptible to severe clinically adverse events and higher mortality from COVID‐19 infection as well as morbidity and mortality from their underlying malignancy. Conclusion: Our review suggests increased risk of mortality and serious clinical events from COVID‐19 infection in cancer patients. However, risk of adverse events does not seem to be increased by cancer therapies. True impact of COVID‐19 on cancer patients will unravel over the next few months. We have also reviewed clinical features of COVID‐19, recent recommendations from various medical, surgical, and radiation oncology societies for major solid tumor types like lung, breast, colorectal, and prostate cancer during the duration of this pandemic.

## Graphical abstract

Our review details the effects of COVID‐19 on clinical outcomes in cancer patients across different continents. COVID‐19 has been associated with higher fatality rate among cancer patients but cancer treatments have not been shown to increase the risk of mortality. We have reviewed the changes recommended in the management of lung, breast, colorectal, and prostate cancer during the duration of the pandemic.

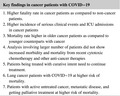

## 1 INTRODUCTION

In December 2019, reports of an atypical pneumonia emerged from the city of Wuhan, Hubei province of China.^1^ The infectious agent was soon identified as a novel enveloped betacoronavirus.^2^, ^3^ Coronaviruses are enveloped non‐segmented positive sense RNA viruses belonging to the family Coronaviridae.^4^ This newly identified coronavirus, has been named Severe Acute Respiratory Syndrome CoronaVirus 2 (SARS‐CoV‐2) by the World Health Organization (WHO). This is the third large scale health crises caused by a betacoronavirus. Severe Acute Respiratory Syndrome CoronaVirus (SARS‐CoV) and Middle East Respiratory Syndrome CoronaVirus (MERS‐CoV) represent the previous health crises. Mortality rate of SARS‐CoV and MERS‐CoV was 10% and 37%, respectively. SARS‐CoV and MERS‐CoV infections were reported in 26 and 27 countries, respectively. A total 10,590 cases of infections were caused by both viruses.^5^, ^6^, ^7^, ^8^, ^9^, ^10^, ^11^ On January 30, WHO declared the novel coronavirus outbreak (2019‐nCoV) a public health emergency of international concern and on March 11, 2020 WHO made the assessment that COVID‐19 can be characterized as a pandemic.^12^ Health crises caused by coronavirus has been unprecedented. COVID‐19 infections which began in Wuhan, China soon spread to other parts of Asia and subsequently to Europe in February 2020. Soon after, Lombardy region of northern Italy was the epicenter of this pandemic. First case in United States was reported on January 20, 2020 of a 35‐year‐old male in Snohomish county, Washington who had recently travelled from Wuhan.^13^ By the end of March, New York city would be the new epicenter of this pandemic. In the subsequent months, COVID‐19 cases soared in South America, affecting Brazil with a rapid pace. As of September 28, 2020, there are 33.1 million cases reported worldwide in 188 countries with 999,369 deaths. In United States alone, there are 7.1 million cases and 204,967 fatalities.^14^ The United States represents approximately 20% share of worldwide coronavirus cases and mortality. Our review article aims at reviewing studies and retrospective analysis of clinical outcomes and treatment related morbidity done on cancer patients with COVID‐19 infection across China, Europe, and North America. We also aim to review the recent recommendations from various cancer societies on treatments of solid tumors during the pandemic.

## 2 MATERIALS AND METHODS

To review the outcomes of cancer patients with COVID‐19, extensive electronic search was undertaken of articles in PubMed database until 21 June 2020. Articles were identified using keywords,” COVID‐19 clinical features” and “COVID‐19 and Cancer.” Articles from China, Italy, United Kingdom, and North America were analyzed. Articles which reiterated the same clinical outcomes were excluded. We used articles in English only. To review the recommendations during COVID‐19 pandemic, we searched guidelines from various international cancer society's such as National Comprehensive Cancer Network (NCCN), European Society of Medical Oncology (ESMO), and American Society for Radiation Oncology (ASTRO) for lung, breast, colorectal, and prostate cancer. Entire texts of the articles reviewed was analyzed and reported.

## 3 CLINICAL FEATURES

Guan et al reviewed the data from 1099 COVID‐19 patients admitted in 552 institutes across mainland China. Positive case was defined as positive result on real‐time reverse‐transcriptase–polymerase‐chain‐reaction (RT‐PCR) assay of nasal and pharyngeal swab specimens. Median age of the patients was 47 years. Median incubation period was 4 days. Fever was predominant symptom noticed in 88.7% patients although only 43.8% had fever at the time of presentation. Second most common symptom was cough seen in 67.8% cases. Shortness of breath was noted in 18.7% cases. 84% (n = 926) were classified as having non‐severe disease and 16% (n = 173) had severe disease. The patients with severe disease were older and had coexisting illnesses. The most common findings on CT chest were ground glass opacities and bilateral patchy shadowing (>50%). 5% patients were admitted to the intensive care unit (ICU), 2.3% required mechanical ventilation, and 1.4% (n = 15) died. Fourteen of the 15 patients who died had severe disease. Ten patients in the whole study were classified as having cancer, with cancer status (stable vs progressive disease) unknown in this study.^15^, ^16^ In‐hospital mortality from COVID‐19 was reported to extremely high from initial studies (up to 28%).^17^ Older age was related to higher mortality. Mortality rate was 8% in people aged 70‐79 years and 15% for patients above the age of 80.^18^ A meta‐analysis of 43 trials which included 3600 patients revealed the overall estimated proportion of severe cases and case‐fatality rate to be 25.6% and 3.6%, respectively.^19^ Studies have revealed human to human transmission through respiratory droplets and through direct contact with infected patients, surfaces and objects. Further studies are underway to determine the risk stratification of different modes of transmission.^20^, ^21^

## 4 CLINICAL OUTCOMES IN CANCER PATIENTS

### 4.1 Outcomes in China

Liang et al reported outcomes on 18 patients with cancer in a prospective cohort of 1590 patients. Most common tumor type was lung cancer (n = 5), followed by colon cancer (n = 4) and breast cancer (n = 3). 25% patients had either chemotherapy or surgery within last 4 weeks. Patients with cancer were older than those without cancer (63.1 vs 48.7 years). Patients with cancer had higher incidence of severe events (ICU admission requiring invasive ventilation) as compared to patients without cancer (39% vs 8%). Patients who recently underwent treatment (chemotherapy or surgery) were also at higher risk of clinical serious events.^22^ Zhang et al reported outcomes from a retrospective case series of 28 cancer patients from three different hospitals in Wuhan, China. Again, most common cancer type was lung cancer (n = 7). Ten patients had metastatic disease. Six patients had undergone some form of cancer treatment (chemotherapy, radiation therapy, targeted therapy, and immune therapy) within the last 14 days of acquiring COVID‐19 infection. Fifteen (53.6%) patients developed serious events (ICU admission, mechanical ventilation, and death) and eight patients died (28.6%). Patients with non‐metastatic disease had less severe events as compared to patients with metastatic disease (44.4% vs 70%). Out of the six patients who had received anti‐cancer treatment within the last 2 weeks, five of them developed severe events.^23^ Dai et al reported data from 105 patients in Wuhan. The analysis included nine patients who had hematological malignancy as well. Like previous studies, Lung cancer was the most common tumor type. Seventeen patients had metastatic cancer. In contrast to prior studies, the median age of both groups (with and without cancer) was similar. Up to 46% patient received cancer treatment (chemotherapy, immunotherapy, radiation, surgery, and targeted therapy with EGFR tyrosine kinase inhibitor) within 40 days of COVID‐19 infection. Patients who received immunotherapy and underwent surgery had higher rates of death and developing critical symptoms. Similar findings regarding developing critical symptoms was not seen in patients undergoing radiation treatment. Like earlier studies, cancer patients were observed to have a higher death rate (OR = 2.34), higher ICU admission rates, and higher chances of needing invasive ventilation. Patients with metastatic cancer had worse outcomes. Interestingly, patients with early non‐metastatic cancer did not differ in outcomes with non‐cancer patients but this finding was not statistically significant. Patients with lung cancer and those with other cancers with lung metastasis also had higher rate of deaths, ICU admission, critical symptoms, and use of mechanical ventilations.^24^ These initial studies pointed toward higher rates of serious clinical events and death in cancer patients. Risk was deemed higher in recently treated patients.

### 4.2 Outcomes in Europe (Italy, United Kingdom)

Following the evolution of the pandemic from China, Europe was affected next by a sudden surge of cases. Among European countries, Italy was the first to be affected severely. By April 1, within a month of first case that was reported in Lombardy region in northern Italy, 100,000 cases and more 12,000 deaths had been reported in the country.^25^ Out of 909 patients who died in the hospital in that registry, 150 patients had active cancer within last 5 years. Lombardy region was the epicenter of the pandemic and accounted for 63.5% deaths across the country.^26^ Tripani et al reported outcomes from a small case series of nine cancer patients with lab confirmed COVID‐19 who were referred to European institute of oncology in Lombardy region. Three were inpatient and six were treated as outpatients. No cancer type was predominant. Eight of the nine patients were actively receiving cancer therapy (n = 4 chemotherapy, n = 2 immunotherapy, and n = 2 small molecules). Three were being treated with curative intent and five patients had metastatic disease. None of the patients required ICU admission, no deaths were reported, and all three inpatients were discharged.^27^

United Kingdom Coronavirus Cancer Monitoring project (UKCCMP) generated database from prospective cohort of 800 patient across 55 cancer centers in the country. Gastrointestinal, respiratory, breast, male genital, and hematological cancers were the most common types (10% or more). Forty‐three percent patients had metastatic disease. Sixty‐five patients had received some form of cancer treatment within the last 4 weeks. More than half of the patients receiving treatment had received cytotoxic chemotherapy. Forty‐five patients had severe or critical infection. Mortality rate was high at 28% (226 out of 800) with 93% of those deaths due to COVID‐19. Patients who died had higher number of co‐morbidities and were older than those who survived. Interestingly, patients who had recently received chemotherapy (within 4 weeks of COVID‐19 infection) did not have increased mortality as compared to those who did not receive chemotherapy. Since the patient receiving chemotherapy were younger (64 years vs 71 years), multivariate analysis was done for age, still no difference in mortality was found. Decreased odds of death were noticed in patients receiving non‐palliative treatment as compared to those receiving palliative treatments. Patients receiving other non‐chemotherapy forms of treatment (immunotherapy, radiation, hormonal, and targeted therapy) were not found to have an additional risk of death.^28^

### 4.3 Outcomes in North America

Surge of cases in Europe was soon followed by rapid increase of cases across the United States in March 2020. New York city became the epicenter of the pandemic soon after. Till date the pandemic has not slowed down as new cases and fatalities continue to mount. Mehta and Goel et al have reported outcomes from New York city hospital on 218 cancer patients with COVID‐19. Seventy‐five patients had solid tumors and 25% had hematological malignancies. Most common tumor type was genitourinary (n = 46) followed by breast (n = 28) and colorectal cancer (n = 21). Total of 61 (28%) patients died. Mortality rate was higher than 50% in patients with lung (55%) and pancreatic cancer (67%). Breast and genitourinary cancer were associated with relatively lower mortality rate (14% and 15% respectively). Among hematological malignancies, myeloid neoplasm had a higher mortality rate than lymphoid tumors. The deceased cohort was older than non‐deceased cohort (76 years vs 66 years). Active chemotherapy and radiation therapy were not associated with increased mortality. Although not statistically significant, active disease (<1 year) and metastatic disease was associated with higher mortality.^29^

COVID‐19 and Cancer Consortium (CCC19) reported outcomes of 928 cancer patients with COVID‐19 infection from United States, Canada, and Spain. Most common tumor type was breast cancer (n = 191) followed by prostate cancer (n = 152). 654 patients had solid tumors, 167 patients had hematological malignancies, and 107 had multiple cancers. Fourty‐five patients were reported to be in remission and 43% had active cancer. Among those with active cancer, 74% had stable or responding cancer, and 26% had progressive cancer. Out of 366 patients on active treatment, 160 received chemotherapy in the last 4 weeks and 206 patients underwent other forms of cancer therapy. Eighty‐nine (10%) patients were given hydroxychloroquine alone, 93 (10%) were given azithromycin alone, 181 (20%) were given a combination of these drugs, and 486 (52%) were treated with neither of these two drugs. Mortality rate was 13% (121 patients died within 30 days of COVID‐19 diagnosis). Interestingly, 59% (n = 71) of the patients who died were never admitted to the ICU. Patients with active cancer had a higher rate of death outside the ICU than those in remission. About 466 (~50%) required admission to hospital, of whom 175 (38%) were actively receiving anti‐cancer therapy. There was an absence of association between 30‐day all‐cause mortality and recent surgery, non‐cytotoxic treatments, and cytotoxic treatment. Another intriguing finding was the strong association of 30‐day all‐cause mortality observed in the group of patients treated with the combination of azithromycin plus hydroxychloroquine (25% mortality) as compared to those treated with hydroxychloroquine alone (12%), with azithromycin alone (13%), and those treated with neither (8%). The combination was more likely to be given to those patients who had severe COVID‐19.^30^ Outcomes have been summarized in Table 1.

**TABLE 1:** Outcomes in cancer patients with COVID‐19

	Country	Number of patients (n)	Type of study	Most common tumor type	Median age (years)	Clinical Outcomes	Adverse outcome with recent cancer treatments^a^
Liang et al^22^	China	18	Prospective cohort	Lung (n = 5)	63.1 (mean age)	Higher risk of serious clinical events, increased risk in those who recently received treatment	Observed
Zhang et al^23^	China	28	Retrospective	Lung (n = 7)	65.0	>50% patients had severe clinical events, Higher risk in metastatic disease patients	Observed
Dai et al^24^	China	105	Prospective cohort	Lung (n = 22)	64.0	Higher death rate in cancer patient's vs non‐cancer pts (OR = 2.34), Higher risk in metastatic disease patients and those who underwent immunotherapy and surgery	Observed
Tripani et al^27^	Italy	9	Retrospective	Urothelial (n = 2)	68.0	3 patients hospitalized, no deaths, no ICU admission	N/A
Lennard et al^28^	United Kingdom	800	Prospective cohort	Gastrointestinal (n = 150)	69.0	28% death rate	Not observed
Mehta and Goel et al^29^	United states of America (US)	218	Prospective	Genitourinary (n = 46)	69.0	28% death rate, case fatality rate higher in hematological malignancy than solid tumors	Not observed
CCC19	US, Canada, Spain	928	Prospective	Breast (n = 191)	66.0	13% mortality, higher rate of death with combination of hydroxychloroquine and azithromycin	Not observed

^a^ Any cancer treatment in last 30 days (surgery, non‐cytotoxic, cytotoxic chemotherapy).

## 5 IMPLICATIONS OF COVID‐19 ON CANCER TREATMENT

### 5.1 Mitigating transmission of SARS‐CoV2 to cancer patients and healthcare workers

Currently, while clinical trials of developing an effective vaccine and treatment are underway, preventing transmission of SARS‐CoV2 remains the desired goal. Prescreening of patients over the phone for symptoms such as fever, cough, shortness of breath, temperature screenings at hospital entry have been employed and is useful.^31^ Physical distancing has been employed in patient waiting area and chemotherapy rooms. Cancer patients, with or without active treatment, have been found to have a higher risk (although small) risk of contracting COVID‐19 as compared to general population. This could be explained due to frequent contact with healthcare system for blood checks, surveillance scans, toxicity checks, and treatments.^32^

Telemedicine should be used when feasible especially for patients who are not on active therapy and those with stable cancer. Patients with symptoms concerning for COVID‐19 need to be isolated from other patients and be tested immediately. In recent weeks, testing capacity has increased manifolds using different tests based on the urgency of getting the results. Testing of patients and healthcare workers with exposure, mild to moderate symptoms with or without fever is essential to ensure continuous delivery of treatments to other patients safely. No visitor policy has been employed as well in outpatient cancer centers and in hospital with few exceptions to help curtail the spread of infection. Non‐emergent routine surgeries were cancelled initially to protect PPE, ICU beds, and develop a surge capacity. Now surgeries have re‐started with pre‐procedure SARS‐CoV2 testing at many centers. Correct hand washing prior to physical examination and use of FDA approved hand‐sanitizers need to be employed by healthcare workers before and after each patient contact. Physical exam only if relevant to patient treatment needs to be performed.

### 5.2 Medical oncology guidelines during COVID‐19

At this time due to rapid evolution of the pandemic and constraints on research and local resources, recommendations have been made by NCCN, ESMO, and ASTRO for most cancer types. Multidisciplinary tumor team discussions are always helpful and recommended. We will review the medical oncology, surgical oncology, and radiation oncology recommendations made for major tumor types reported in clinical studies reviewed earlier in this article. ESMO guidelines prioritizes patients into high priority (immediate intervention, clinically unstable), medium priority (situation non‐critical but delay beyond 6 weeks could potentially impact outcomes), and low priority (patient is stable and services can be delayed during the duration of the pandemic).^33^

#### 5.2.1 Lung cancer

Curative intent adjuvant chemotherapy needs to continue as planned. However, if resources are limited or patient prefers to delay treatment due to COVID‐19, then adjuvant therapy can be delayed up to 4 months.^34^ For stage 3 non‐small cell lung cancers (NSCLC) undergoing concurrent definitive chemoradiation (chemo‐RT), immunotherapy can be delayed for 6 weeks after chemo‐RT. Extended interval immunotherapy can be used (every 6 week vs 3 week dosing of pembrolizumab, every 4 week vs 2 week nivolumab). Extended intervals between restaging or surveillance scans is also recommended. Use of oral agents is preferred over intravenous chemotherapy if possible. Use of every 12‐week zoledronic acid in patients on oral targeted therapy instead of every 4 weeks should be considered especially if they have been treated for over 12 months. Discussion of goals of care and code status with the patient is of paramount importance given high risk of mortality from COVID‐19 infection in patients with lung cancer.^35^ ESMO recommends neoadjuvant chemotherapy for stage 2 lung cancer, thus, enabling deference of surgery by 3 months.^36^

#### 5.2.2 Breast cancer

Neoadjuvant treatment can be preferred in cases where both neoadjuvant therapy or surgery first are both options to save surgical, personnel, and hospital resources. Patients who are already on neoadjuvant therapy should continue the treatment per protocol. Docetaxel which can is given every 3 weeks can be used instead of weekly paclitaxel if feasible after discussing with the patient on side‐effects and benefits of both drugs to minimize weekly visit to the cancer center. Patient with hormone positive, human epidermal growth factor receptor 2 (HER 2) neu negative tumors can receive 6‐12 months of neoadjuvant endocrine therapy if there is no disease progression on treatment. Triple negative breast cancer needs to be treated with standard chemotherapy regimens. Patients with metastatic breast cancer who are stable on single agent chemotherapy can be managed with telemedicine and their restaging scans deferred for a short interval. Oral agents in select settings are preferred. Bisphosphonates and denosumab can be deferred in patients who do not have hypercalcemia for a short interval to decrease visits. LHRH agonists 3 monthly doses can be used instead of monthly injection. G‐CSF can be used more liberally and for regimens with intermediate risk of neutropenia (10‐20%) to reduce risk of neutropenia and hospitalization.^37^

#### 5.2.3 Colorectal cancer

NCCN and ESMO recommends the use of oral agents such as capecitabine over 5‐FU whenever feasible which allows for treatment at home. Consider capecitabine 1 week on 1 week off dosing for 28‐day cycle because of reduced need for intensive monitoring and lower toxicity. For stage 3 patients with T1, T2, or T3 and N1 cancers, using 3 months capecitabine‐oxaliplatin (Cape‐Ox) had similar disease‐free survival similar to 6 months of Cape‐Ox (IDEA trial) and can be used during the pandemic.^38^ Patient with stage 2 or 3 colon cancer, unable to get surgery can be treated with neoadjuvant chemotherapy as a bridge to surgery. Holding surveillance colonoscopies is also recommended. For stage 4 colon or rectal cancer, prefer using Cape‐Ox every 21 days instead of 5‐FU/Oxaliplatin regimen every 14 days to reduce the visits to cancer center. G‐CSF given at home is preferred than giving it the next day at cancer center. Tumors which are MMR‐deficient/MSI‐high use of immunotherapy is advocated instead of cytotoxic chemotherapy.^39^ Immunotherapy doses used in extended intervals dosing are preferred. Central venous catheter flushes can be done at 12‐week intervals. Patients with stage 4 disease, especially those who are older and have multiple co‐morbidities should have a goal of care and do not resuscitate discussions.^40^

### 5.3 Prostate cancer

Patients with very low, low, and favorable intermediate risk prostate cancer, their work up can be deferred until it is deemed safe. Patients with non‐metastatic disease and PSA doubling time >9 months, their androgen deprivation therapy (ADT) can be deferred for some time. Asymptomatic patients with unfavorable intermediate risk or high risk can be started on neoadjuvant ADT and their definitive radiation therapy can be deferred for 4‐6 months. It is advisable to use 3, 4, or 6 months dosing of ADT rather than monthly. Defer the use of Sipuleucel‐T in patients with advanced disease until risk of COVID‐19 resolves in select cases.^41^ Prefer using androgen receptor targeted agents such as Abiraterone acetate and Enzalutamide in place of cytotoxic chemotherapy when possible for patients with metastatic hormone sensitive and metastatic castration resistant prostate cancer.^42^ Physicians can consider using Enzalutamide as it does not require concomitant steroids like Abiraterone acetate during the duration of pandemic.

### 5.4 Surgical oncology guidelines during COVID‐19

Elective surgeries were deferred in the beginning of the pandemic in United States to conserve ICU resources and focus on planning for COVID‐19 surge. If resources are available, all surgeries with curative intent should be done as planned. However, if resources are limited then certain recommendations can be followed on a case to case basis.

#### 5.4.1 Lung cancer

Most lung cancer surgeries represent emergent surgeries that cannot be delayed over a long period of time. Surgeries with curative intent should proceed as normal. Pre‐procedure SARS‐CoV2 testing prior to any cancer surgery is recommended if testing resources permit. In limited resources, Stage 1 lepidic adenocarcinoma and stage 1A1 can be deferred for 3 months. Surgery for stage 1A2, Stage 2, and stage 3A (non‐N2) NSCLC are considered urgent. Stage 3A NSCLC (T1‐2, N2) which are deemed surgically resectable can receive neoadjuvant induction chemotherapy or concurrent chemoradiation followed by restaging scans and surgery.

#### 5.4.2 Breast cancer

NCCN encourages utilizing neoadjuvant chemotherapy or endocrine therapy in patients who qualify. Elective mastectomy should be discouraged in patients who are candidates for breast conservation surgery. In patients who require a mastectomy, reconstruction with implants or tissue expanders should only be performed if adequate resources are available. Autologous reconstruction should be deferred.^37^, ^43^

#### 5.4.3 Colorectal cancer

Society of surgical oncology (SSO) has given recommendations based on 3 phases of the pandemic surge. Phase 1 is pre‐surge (few COVID patients, institutional resources available), phase 2 early surge (COVID patient number acceleration, limited institutional resources), and phase 3 surge/peak (all institutional resources focused on COVID‐19, no ICU/OR resources available). Surgeries for intestinal perforation, complete bowel obstruction, or acute hemorrhage from the cancer are considered emergent and cannot be deferred in any phase. Similarly, neoadjuvant chemotherapy for locally advanced colon cancer can be considered. Also consider total neoadjuvant therapy including chemoradiation followed by systemic chemotherapy prior to surgical resection of locally advanced rectal cancer. Always consider transferring patient to another center who has available resources and capacity. In phase 1, surgeries for benign and malignant colorectal polyps along with prophylactic procedures for hereditary and inflammatory conditions may be deferred. Curative intent surgeries can be undertaken for non‐metastatic colon and rectal cancer after neoadjuvant therapy. In phase 2, surgery for colorectal cancers which are minimally symptomatic or asymptomatic may have to be deferred. Emergent surgeries, significantly symptomatic, bleeding and obstruction colorectal tumors can be taken for surgery. In phase 3, only emergent life‐threating surgeries would be considered.^44^

### 5.5 Prostate cancer

NCCN states that surgeries for unfavorable intermediate risk, high risk, very high‐risk prostate cancer can be deferred for 6 months within minimal impact on outcome based on results from John Hopkins group.^41^, ^45^

### 5.6 Radiation oncology guidelines during COVID‐19

Adapting to the challenges of delivering radiation therapy during the COVID‐19 pandemic has led to the development of multiple guidelines from professional societies and individual institutions. One challenge unique to radiation therapy is the delivery of daily fractionated treatment over a prolonged period of time. Each fraction poses another risk of exposure to healthcare environment, and thus the focus has been placed on either delaying treatment when safe or hypofractionating treatment regimens as much as is safely possible.

#### 5.6.1 Lung cancer

A joint panel was convened by ASTRO and the European Society for Radiotherapy and Oncology (ESTRO) to determine consensus recommendations for the treatment of a variety of lung cancer scenarios. The panel addressed both delaying radiation as well as hypofractionation schemes. For common lung cancer scenarios such as Stage 3 NSCLC, Limited Stage Small Cell Lung Cancer (LS SCLC) and palliative treatment of NSCLC, there was strong consensus to proceed to treatment without delay. For Post‐Operative Radiation Therapy (PORT) for NSCLC and prophylactic cranial irradiation (PCI) for LS SCLC, there was strong consensus to delay the initiation of treatment in the setting of the COVID‐19 pandemic. In Stage I NSCLC, the respondents split evenly on whether or not to delay treatment and additionally were split on stereotactic body radiation therapy (SBRT) fractionation, namely utilizing conventional 3‐4 treatment versus a single fraction of 30‐34 Gy. Similarly, for Stage 3 NSCLC, there was no clear recommendation for hypofractionation. However, standard fractionation (30‐33 fractions) was recommended in the setting of concurrent chemoradiation. Standard fractionation was also recommended for PORT, LS SCLC, and PCI. Hypofractionation, delivering 1, 2, or 5 fractions was recommended in the palliative setting for NSCLC over traditional 10 fraction courses.^46^

#### 5.6.2 Breast cancer

In a similar fashion to the lung cancer recommendations above, Coles et al. published a clear set of international guidelines for the treatment of breast cancer during COVID‐19. In addition to recommendations on treatment delay and hypofractionation, recommendations were made on modifying treatment fields to minimize treatment complexity to ease strain on the radiation therapy team.^47^ Based on the PRIME II Trial, omission of radiation was recommended in women over 65 years of age with tumors up to 30 mm, low to moderate grade, node negative, estrogen receptor (ER) positive, HER 2 neu negative who will be receiving endocrine therapy.^48^ For women with node negative breast cancer who do require radiation therapy, the guidance suggested was to use a 5 fraction treatment regimen.^49^, ^50^ A similar recommendation to utilize hypofractionation was made for patients requiring chest wall or regional nodal radiation therapy, although more moderate hypofractionation schemes, such as 40 Gy in 15 fractions, were recommended.^51^, ^52^ In regards to reducing the complexity of treatment, in women over 40 without significant risk factors for local recurrence, omitting the tumor bed boost was recommended. In post‐menopausal women with favorable disease characteristics and 1‐2 lymph node macrometastases, omission of nodal radiation therapy was recommended.47 Braunstein et al published similar guidelines that are included on the ASTRO COVID‐19 Clinical Guidelines. The recommendations come from a single, high volume comprehensive cancer center and identify groups of women who could omit or delay radiation, appropriate hypofractionation regimens and omission of a tumor bed boost.^53^

#### 5.6.3 Colorectal cancer

While radiation therapy plays a very limited role in colon cancer, it is a mainstay in the treatment course of rectal cancer. As with the other disease sites reviewed, an international consensus guideline publication by Marijnen et al very nicely compares standard ESMO guidelines to treatment recommendations during COVID‐19. Omitting pre‐operative radiation in the treatment of early stage disease remains unchanged. In the intermediate risk group (T3 any N or T1‐2N1‐2), the recommendation from the European community is Total Mesorectal Excision (TME) alone, or short course radiation therapy followed by surgery if quality surgery cannot be guaranteed. However, NCCN guidelines in the US recommend neoadjuvant therapy followed by surgery for this subgroup of patients and SSO recommends neoadjuvant chemo‐RT followed by systemic chemotherapy followed by surgery for similar patients. Multidisciplinary discussion is, thus, recommended prior to embarking treatment for such patients who are treated with curative intent. Similar neoadjuvant recommendations also exist for more advanced stage disease in the NCCN guidelines, with combined chemotherapy and long course radiation therapy or short course radiation therapy +/− 12‐16 weeks of chemotherapy.^54^ In the COVID‐19 setting, the Marijnen group recommends short course radiation therapy with a planned delay in taking the patient to surgery. De Felice et al weigh in on the short course versus long course radiation regimens. The recommendation is that in the setting of the COVID‐19 pandemic, short course radiation therapy (25 Gy in 5 fractions) with a 5‐13 week delay to surgery may be the most appropriate option.^55^

#### 5.6.4 Prostate cancer

An excellent international consensus paper by Zaorsky et al details recommendations for prostate cancer patients during the COVID‐19 outbreak. The recommendations followed the RADS framework of Remote visits, Avoid radiation, Defer radiation, and Shorten radiation. For low risk prostate patients, active surveillance (AS) was the preferred treatment modality. Similarly, for favorable intermediate risk patients, AS was preferred during the pandemic with radiation therapy delayed until safe. For unfavorable intermediate risk, high risk, and node positive patients, ADT can be utilized to delay radiation therapy 4‐6 months. Once treatment is initiated for these patients, a hypofractionated approach of 5‐20 fractions is recommended (as compared to standard fractionation of 40+ fractions). In the postoperative setting, early salvage radiation is preferred to adjuvant radiation. Radiation is recommended using a 20 fraction regimen +/− ADT, with androgen deprivation allowing for a 4‐6 months delay in the start of treatment. As with the patient groups mentioned above, in the setting of oligometastatic or low volume metastatic disease, ADT can be utilized to delay radiation up to 6 months. Radiation therapy in this metastatic setting should be delivered in 1‐6 fractions. These guidelines also champion the use of telehealth visits for prostate cancer patients during the pandemic, with the benefit of digital rectal exam noted to be less than the associated risk of a clinic visit.^56^ Key treatment recommendations have been summarized in Table 2.

**TABLE 2:** Treatment recommendations during COVID‐19 Pandemic

	Cancer type	Recommendations during COVID−19 pandemic^a^
Medical Oncology		
	Lung	Continue curative intent chemotherapy Stage 3‐can wait for 6 weeks after chemo‐RT to start immunotherapy Consider extended interval immunotherapy^b^ Extended interval between restaging scans^b^ Use G‐CSF for intermediate risk chemotherapy regimen^b^ Consider neoadjuvant chemotherapy for stage 2 lung cancer Timely Discussion of goals and care^b^
	Breast	Prefer use of neoadjuvant chemotherapy if indicated Prefer use of neoadjuvant hormonal therapy if indicated Consider using every 3 weeks docetaxel instead of weekly paclitaxel Patients already on neoadjuvant therapy, continue per protocol Use telemedicine whenever feasible^b^ LHRH agonists every 3‐month dosing instead of monthly
	Colorectal	Use oral chemotherapy agents whenever possible^b^ T1, T2, T3 N1 cancer Use Cape‐Ox for 3 months Use neoadjuvant chemotherapy for stage 2/3 cancers as a bridge to surgery Prefer Cape‐Ox over FOLFOX for stage 4 disease G‐CSF at home preferred^b^ Central venous catheter flushes every 12 weeks instead of monthly^b^
	Prostate	Consider using Abiraterone or Enzalutamide over cytotoxic chemotherapy if possible Use 3, 4, 6‐monhtly ADT doses
Surgical Oncology		
	Lung	Continue curative intent lung cancer surgeries Pre‐procedure SARS‐COV2 testing is recommended^b^ Stage 1 lepidic adenocarcinoma and stage 1A1 can be deferred for upto 3 months Consider neoadjuvant chemo‐RT or induction chemotherapy for surgically resectable stage 3 lung cancer
	Breast	Encourage breast conservation surgery in patients who are candidates In patients undergoing mastectomy, perform reconstruction with implants or tissue expanders if resources are adequately available Autologous reconstruction should be deferred
	Colorectal	Emergent surgeries (intestinal perforation, complete bowel obstruction, acute hemorrhage) cannot be deferred Consider neoadjuvant chemotherapy or chemo‐RT when indicated Consider transferring patients to institutes where resources are available^@^ Phase 1: Defer surgeries for benign, malignant colorectal polyps and prophylactic procedures for hereditary and inflammatory conditions Phase 2: May defer surgery for minimally symptomatic or asymptomatic cases Phase 3: Consider only emergent and life‐threatening surgeries
	Prostate	Surgeries for unfavorable intermediate risk, high risk, very high‐risk prostate cancer can be deferred for 6 months within minimal impact on outcome
Radiation Oncology		
	Lung	Continue standard of care for Stage III NSCLC and LS‐SCLC Delay PORT and PCI Case by case assessment of delay/fractionation scheme for Stage I NSCLC Hypofractionation for palliation, but no clear consensus on hypofractionation for definitive treatment
	Breast	Omit radiation in women over 65 with early stage breast cancer with favorable tumor characteristics For node‐negative women who require whole breast radiation, utilize a 5 fraction regimen Use hypofractionation for chest wall or regional nodal radiation (15‐16 fractions) Omit tumor bed boost in women over 40 without significant risk of local recurrence
	Colorectal	Favor short course (5 fraction) neoadjuvant radiation in rectal cancer. Plan to delay surgery following short course radiation
	Prostate	Active surveillance for low and favorable intermediate risk patients Use ADT to delay radiation 4‐6 months in unfavorable intermediate, high risk and node positive patients and use a hypofractionated radiation course of 5‐20 fractions Favor early salvage radiation over adjuvant treatment and utilize a hypofractionated regimen
Mental health and wellbeing		Acknowledgement how patients are feeling anxious Promote physical fitness at home Promote positive joyous activities Promote spending time in nature Encourage connecting with family and friends virtually

^a^ Decisions to be made by treating physician and on case to case basis.

^b^ Applicable to other cancer types as well.

^@^ If hospital or institutional resources are extremely limited and COVID‐19 cases have surged.

#### 5.6.5 Novel utilization of radiation therapy.

In addition to adapting oncologic treatments during the pandemic, radiation oncologists are exploring methods to use radiation in the fight against COVID. Currently, the idea of low‐dose whole lung irradiation is being explored in the clinical trial setting.^57^, ^58^, ^59^, ^60^ Harkening back to data from roentgen therapy for pneumonia at the beginning of the prior century, low doses of radiation (less than 1 Gy) are thought to have anti‐inflammatory properties related to inhibition of pro‐inflammatory cytokines.^61^ This novel (in the modern era) use of low dose, whole lung radiation holds the promise of mitigating some of the life threatening symptoms of COVID‐19.

### 5.7 Mental health and well‐being

COVID‐19 pandemic has negatively impacted the mental health of cancer patients as well. It is causing stress, severe anxiety from fear of infection and a decrease in social support from family members especially for patients residing in nursing homes.

There is not much literature on consequences of epidemic and physical distancing on mental health issues. However, numerous publications on past traumatic (World Trade 9/11 attack, various mass shootings), natural (earthquake, hurricane) reported increased risk of anxiety, post‐traumatic stress disorder (PTSD), depression, substance use disorders, and behavioral issues (child abuse, domestic violence).^62^ Statistics showed 25% of New York city population increased use of alcohol and 10% New Yorkers had increased depression symptoms after the 9/11 attack.^63^, ^64^ There was an increase in psychological distress, PTSD, and stress in communities during SARS epidemic.^65^ Increase in stress associated symptoms like insomnia, irritability, fear, boredom, anxiety, decreased perception of safety, anger, frustration, scapegoating, and increased presentation to healthcare due to fear of illness.^66^ Some patients will require mental health evaluation and care, while others may need supportive therapy to promote well‐being and safety. Considering serious cancer illness along with numerous uncertainties surrounding this pandemic, evaluation of suicidality, and immediate mental health consultation or emergency psychiatric hospitalization should be considered. In addition, providers should consider recommendations by NCCN for promoting mental well‐being doing COVID‐19 pandemic in cancer patients such as acknowledging and validating the mix of emotions the patients are feeling, promoting physical fitness activities at home, advocating connecting with nature during self‐isolation, encourage connecting with friends and family virtually, and doing things that one enjoys.^67^

## 6 DISCUSSION

Studies from China, Europe, and North America have consistently shown that cancer patients with COVID‐19 have a higher risk of mortality, increased risk of ICU admission, and severe clinical events when compared to non‐cancer patients. Recent meta‐analysis has revealed all‐cause mortality was higher in cancer patients versus non‐cancer patients (2,034 deaths; RR, 1.66; 95% CI, 1.33 to 2.07; *p* < .0001; eight studies with 37,807 patients). The need for ICU admission was also more likely in patients with versus without cancer (3,220 events; RR, 1.56; 95% CI, 1.31 to 1.87; *p* < .0001; 26 studies with 15,375 patients).^68^ Similar findings were observed in previous coronavirus outbreaks of MERS with mortality rate being 84% in cancer patients and 39% in non‐cancer patients.^69^ Cancer patient population is a vulnerable patient group that is at an increased risk likely due to relatively older age, poor performance status either due to cancer or anti‐cancer, as well as immunosuppression that occurs in inherently in some hematological malignancies. Until an effective treatment and a vaccine is available, prevention of SARS‐CoV2 cancer patients should be the goal. Cancer patients need to get other vaccinations (influenza, pneumococcal, meningococcal disease) done to prevent or mitigate risk of other infections. Hospitals and Cancer treatment centers need to follow strict infection control guidelines and physical distancing between patients along with protecting healthcare workers in close contact with cancer patients.

Lung cancer is a common malignancy across the world and is extremely lethal. This is due to presence of metastasis at the time of diagnosis and early recurrence despite the use of chemotherapy and radiation therapy. Lung cancer was also the most common type of cancer in COVID‐19 patients in China. Mortality in lung cancer patients was extremely high in all studies. Bronchogenic carcinoma patients represent a very high‐risk group due to pre‐existing lung damage from the disease, decreased ventilatory functions due to pre‐existing co‐morbidities like COPD, history of smoking and smoking induced other co‐morbidities like coronary artery disease.

Initial studies done in China (Liang et al, Zhang et al) pointed toward increased mortality and severe clinical events in patient who recently underwent chemotherapy.^22^, ^23^ However, studies done in United States and United States with larger number of patients did not reveal high risk of death or severe events in the same population.^28^, ^29^, ^30^ Currently, we do not have enough evidence to definitively link chemotherapy or other therapies with increased risk of death. At this time, it is recommended to defer treatment by 14 days in patients who test for SARS‐CoV2 if possible and patients should be symptom free, fever free without antipyretics for at least 3 days before getting treated for their cancer. Their treatment should be delivered at an isolated area, away from main infusion area and should have 2 negative SARS‐CoV2 tests done at least 24 h apart.^70^

Median age of COVID‐19 patients in initial studies was 47‐49 years of age. Median age in cancer patients reviewed in this article (reviewed in Table 1) is in their sixties.^15^, ^16^ In China, Europe, and North America advanced age has been a main risk factor for increased mortality in COVID‐19 patients.^71^, ^72^, ^73^ Similar findings have been seen in patients with cancer with age being a major factor in increased mortality and risk of severe clinical events. Older cancer patients with COVID‐19 were at a higher risk of mortality as compared to younger cancer patients with COVID‐19 and with patients in the same age group without cancer.^22^, ^24^, ^29^, ^30^ However, in contrast to results from earlier studies, recent meta‐analysis has shown elderly patients with cancer may not be at increased risk of death when infected with COVID‐19 as compared to their counterparts without cancer.^68^

Dai et al noted in their prospective cohort of 105 cancer patients in which six patients who received immunotherapy within last 40 days had a higher rate of death and rate of developing critical symptoms as compared to other cancer treatment modalities.^24^ Lucia Bonomi et al also reported of a case of stage 4 lung adenocarcinoma on treatment with nivolumab, anti PD‐1 (programmed cell death 1) agent, for 5 years having stable disease who died of COVID‐19 within 5 days of diagnosis.^74^ Use of immunotherapy using anti PD‐1 and anti PDL‐1 (programmed cell death ligand 1) agents has resulted in significant improvements in quality of life and survival for patients with various forms of malignancies. This has been noted very heavily in patients with lung cancer. These treatments are considerably easier to tolerate than conventional cytotoxic chemotherapy. However, these agents are associated with various immune related adverse events pneumonitis being one of them. Immunotherapy agents work by unleashing antitumor immune response to achieve long terms tumor growth control.^75^ Patients with severe COVID‐19 admitted to the intensive care unit were more likely to have proinflammatory cytokines such as IFN‐γ, IP‐10, MCP‐1, IL‐1β, IL‐4, and IL‐10.^16^, ^76^ It is difficult to make conclusions whether immunotherapy can adversely affect outcomes in cancer patients with COVID‐19 based on small number of patients reported so far. It is prudent that this aspect of cancer treatment be evaluated through more studies as the use of immunotherapy has increased in the last few years considerably and the improvement in prognosis with use of these agents has been considerable at the cost of minimal side‐effects. Key findings of this review article have been summarized in Table 3.

**TABLE 3:** Key Findings of COVID‐19 among cancer patients

Key findings in cancer patients with COVID−19
1. Higher fatality rate in cancer patients as compared to non‐cancer patients. 2. Higher incidence of serious clinical events and ICU admissions in cancer patients 3. Mortality rate higher in older cancer patients as compared to younger counterparts with cancer 4. Analysis involving larger number of patients did not show increased morbidity and mortality from recent cytotoxic chemotherapy and other anti‐cancer therapies 5. Patients being treated with curative intent need to continue treatment. 6. Lung cancer patients with COVID−19 at higher risk of mortality. 7. Patients with active untreated cancer, metastatic disease, and getting palliative treatment at higher risk of mortality.

## 7 CONCLUSION

SARS‐CoV‐2 is a novel coronavirus to which global population has had no prior exposure. The medical, economic, and social impact of COVID‐19 pandemic has been devastating. Its adverse effect on cancer patients has been amplified due to their older age, interruption of treatments, immunosuppressed condition due to pre‐existing cancer and toxic therapies. Research to develop a safe, effective vaccine and treatment agents which can reduce morbidity and mortality of the infection is ongoing. At present time, for cancer patients, prevention is better than cure. Mitigation of the spread of the virus is key in preventing excess fatalities among cancer patients. Cancer institutions must take all measures to prevent the spread of COVID‐19 infection within treatment centers. Oncologists need to continue therapies aimed at curing and treating cancer despite the pandemic using recommendations from various professional societies as mortality from untreated active malignancy is extremely high. The true impact of COVID‐19 on clinical outcomes is yet to be determined as patients have missed appointments, screening procedures, imaging studies, and most importantly their treatments.

## CONFLICT OF INTEREST

Authors have no conflict of Interest.

## AUTHOR CONTRIBUTION

We confirm that the manuscript is original work/research. We confirm that this manuscript has not been published and is not under consideration elsewhere. Ankit Madan conceptualized the review article. Ankit Madan contributed to medical oncology and surgery recommendations. Joshua Siglin contributed radiation oncology recommendations. Aleem Khan contributed mental health recommendations. All authors contributed in reviewing the manuscript and approved the final version of the manuscript.

## Data Availability

This is a review article. The data discussed in this study is publicly available.
